# Association between Dietary Magnesium Intake and Hyperuricemia

**DOI:** 10.1371/journal.pone.0141079

**Published:** 2015-11-04

**Authors:** Yi-lun Wang, Chao Zeng, Jie Wei, Tuo Yang, Hui Li, Zhen-han Deng, Ye Yang, Yi Zhang, Xiang Ding, Dong-xing Xie, Tu-bao Yang, Guang-hua Lei

**Affiliations:** 1 Department of Orthopaedics, Xiangya Hospital, Central South University, Changsha, Hunan Province, 410008, China; 2 Department of Epidemiology and Health Statistics, School of Public Health, Central South University, Changsha, Hunan Province, 410008, China; IPK, GERMANY

## Abstract

**Objective:**

To examine the cross-sectional associations between dietary magnesium (Mg) intake and hyperuricemia (HU).

**Methods:**

5168 subjects were included in this study. Dietary intake was assessed using a validated semi-quantitative food frequency questionnaire. Hyperuricemia (HU) was defined as uric acid ≥ 416 μmol/L for male population and ≥ 360 μmol/L for female. A multivariable logistic analysis model was applied to test the associations after adjusting a number of potential confounding factors.

**Results:**

The relative odds of the overall prevalence of HU were decreased by 0.57 times in the fourth quintile of Mg intake (OR 0.57, 95% CI 0.35–0.94) and 0.55 times in the fifth quintile (OR 0.55, 95% CI 0.30–1.01) comparing with the lowest quintile, and *P* for trend was 0.091. The results of multivariable linear regression also suggested a significant inverse association between serum uric acid and Mg intake (β = -0.028, P = 0.022). For male, the relative odds of HU were decreased by 0.62 times in the third quintile of Mg intake (OR 0.62, 95% CI 0.40–0.97), 0.40 times in the fourth quintile (OR 0.40, 95% CI 0.23–0.72) and 0.35 times in the fifth quintile (OR 0.35, 95% CI 0.17–0.71) comparing with the lowest quintile, and *P* for trend was 0.006. Multivariable adjusted inverse association was also existed between serum uric acid and Mg intake in male population (β = -0.061, P = 0.002). However, no significant association was observed between dietary Mg intake and HU for female.

**Conclusions:**

The findings of this cross-sectional study indicated that dietary Mg intake is inversely associated with HU, independent of some major confounding factors. In addition, this association remains valid for the male subgroup, but not for the female subgroup.

**Level of Evidence:**

LevelIII, cross-sectional study.

## Introduction

Hyperuricemia (HU) is a major cause of disability, which receives increasing attention in recent decades because of its high prevalence in the global context [1–3]. Epidemiological data showed that about 21% of American adults suffer from HU [4], and in some Asian countries, the prevalence of HU is ranged from 13% to 25.8% [5–8]. Emerging data indicated that HU can increase the risk of hypertension, cardiovascular disease, diabetes and chronic kidney disease [9–13]. Meanwhile, some common disorders, such as dyslipidemia and hyperglycemia, have also been reported to be positively correlated with the uric acid level in serum [14–16]. However, the specific pathogenesis of HU has not yet been fully elucidated.

Magnesium (Mg) is an essential nutrient for humans. Nielsen [17] suggested that the marginal-to-moderate Mg deficiency could exacerbate chronic inflammatory stress and thus contribute to the increased risk of chronic diseases such as atherosclerosis, hypertension, osteoporosis, diabetes, and cancer. Emerging data revealed that people with low dietary Mg intake were associated with the high serum C-reactive protein (CRP) status, which is the most sensitive biomaker for inflammation [18–22]. In addition, several studies have demonstrated that the occurrence of acute inflammation in gout could be strongly predicted based on the degree of HU [23–25]. In view of these factors, Mg deficiency may be related to HU to a certain extent. However, there was only one study showing an inverse relationship between Mg deficiency and the increased serum uric acid level among 94 diabetic retinopathy patients [26]. To our best knowledge, there is not yet a study conducted on a large sample that directly examined the association between dietary Mg intake and the prevalence of HU. The purpose of this cross-sectional study was to explore the aforementioned association based on the following hypothesis: dietary Mg intake is negatively associated with the prevalence of HU.

## Materials and Methods

### Study population

This cross-sectional study was conducted in the Department of Health Examination Center Xiangya Hospital, Central South University in Changsha, Hunan Province, China. We obtained approval for this study from the ethics committee at Xiangya Hospital, Central South University. Also, we obtained written informed consent from the subjects in our study. The Xiangya Hospital Health Management Center Study (XYHMCS) included a cohort consisting mainly of apparently healthy Chinese people from general public for health screening. This overall XYHMCS mainly aimed to explore the risk factors (e.g., dietary factors, serum micronutrients level, lifestyle behaviors) of various diseases, such as HU, osteoarthritis, and so on. The study design has been published previously [27]. Routine health checkups are very common in China, because the Chinese government encourage people to take periodic medical examinations. Registered nurses interviewed all subjects during the examination using a standard questionnaire, with the purpose to collect information on demographic characteristics and health-related habits. Subjects were selected according to the following inclusion criteria: 1) 40 years old or above; 2) undergoing serum uric acid measurement; 3) completion of the semi-quantitative food frequency questionnaire (FFQ) about the average consumption of foods and drinks over the past 1 year; 4) availability of all basic characteristics, including age, gender, body mass index (BMI), smoking status, alcohol drinking status, etc. In the beginning, this cross-sectional study included 10387 subjects who were undergoing routine checkups including serum uric acid measurement at the Department of Health Examination Center Xiangya Hospital, Central South University in Changsha, Hunan Province, China, from October 2013 to July 2014. Then, 9420 individuals were over 40 years old, and 9402 of them were available for basic characteristics, such as BMI. Eventually, 5168 subjects completed the FFQ, and the overall response rate of the survey was 54.9%. There was no significant difference between participants who completed and who did not complete the FFQ in terms of the prevalence of HU in male, female and total population, and the characteristics including age and BMI.

### Assessment HU

All blood samples were drawn after a 12-hour overnight fast and were kept at 4°C until analysis. Uric acid was detected on Beckman Coulter AU 5800 (Beckman Coulter Inc., Brea, CA, USA). HU was defined as uric acid ≥ 416 μmol/L for male and ≥ 360 μmol/L for female.

### Assessment of dietary and non-dietary exposures

Dietary intake was evaluated by using a semi-quantitative food frequency questionnaire (SFFQ) which was specially designed for the population in Hunan province of China. This SFFQ contains 63 food items which are popularly consumed in Hunan province. Subjects were requested to answer how frequently (never, once per month, two to three times per month, one to three times per week, four to five times per week, once per day, twice per day, or three times and above per day) they consumed each food item in the past year. There are 6 options for the average amount of food consumption in each time: less than 100g, 100–200g, 201–300g, 301–400g, 401g-500g, and more than 500g. Color pictures showing food samples with labeled weights were given to participants as a reference. The SFFQ was answered in a self-administered way or completed through interviews by professional researchers. The validity of the SFFQ was tested through comparing with the 24-hour dietary recall method, where the tested samples were randomly selected from the same study population. The correlation coefficient between the SFFQ and the 24-h recall was 0.53 for the measurement of Mg intake. The Chinese Food Composition Table [28] was referenced to calculate the individual composition of macronutrients and micronutrients of the included foods. The recommended daily allowance of Mg was 350 mg/day according to the Chinese Food Composition Table. This SFFQ has been validated and used in a previous published study [29].

The weight and height of each subjects were measured respectively to calculate the BMI. Participants were also asked about their average frequency of physical activity (never, one to two times per week, three to four times per week, five times and above per week) and average duration of physical activity (within half an hour, half an hour to one hour, one to two hours, more than two hours). The smoking and alcohol drinking status were asked face to face. The blood fasting glucose, high density lipoprotein cholesterol (HDL- cholesterol), low density lipoprotein cholesterol (LDL- cholesterol) and triglyceride (TG) were also detected on Beckman Coulter AU 5800 (Beckman Coulter Inc., Brea, CA, USA). Blood pressure was measured using an electronic sphygmomanometer. Subjects with the fasting glucose ≥ 7.0 mmol/L or currently undergoing drug treatment for blood glucose control were regarded as diabetes patients, and subjects with the systolic blood pressure ≥ 140 mm Hg or diastolic blood pressure ≥ 90 mm Hg or currently using antihypertensive medication were regarded as hypertension patients.

### Statistical analysis

The quantitative data are expressed as mean ± standard deviation, and the qualitative data are expressed in percentage. The Mg intake was classified into five categories based on the quintile distribution: ≤ 209.42, 209.43–294.56, 294.57–383.53, 383.54–535.16 and ≥ 535.17 mg/day in total population; ≤ 240.50, 240.51–322.54, 322.55–421.74, 421.75–561.69 and ≥ 561.70 mg/day in male population; and ≤ 188.31, 188.32–263.14, 263.15–345.95, 345.96–493.10 and ≥ 493.11 mg/day in female population. Differences in continuous data were evaluated by the one-way classification ANOVA (normally distributed data) or the Kruskal-Wallis H test (non-normally distributed data), while differences in qualitative data were assessed by the χ^2^ test. The odds ratios (ORs) with 95% confidence intervals (CIs) for the association between HU and dietary Mg were calculated for each quintile of Mg intake, respectively, and the quintile with the lowest value was regarded as the reference category. In order to calculate the adjusted OR of each quintile of Mg intake, a multivariable model were adopted in the logistic analyses for total population, male population and female population, respectively, shown in Table 1. Covariant variables includes age, BMI, gender, educational level, activity level, total energy intake, fiber intake, smoking status, alcohol drinking status, nutrients supplementation, diabetes, hypertension, serum creatinine, HDL-cholesterol, LDL-cholesterol and TG. Tests for linear trends were conducted based on logistic regression using a median variable of Mg level in each category. Multivariable linear regression was also conducted to evaluate the association between serum uric acid and Mg intake after adjusted for age, BMI, gender, educational level, activity level, total energy intake, fiber intake, smoking status, alcohol drinking status, nutrients supplementation, diabetes, hypertension, serum creatinine, HDL-cholesterol, LDL-cholesterol and TG. All data analyses were performed using SPSS 17.0; a *P* value equal to or less than 0.05 was considered to be statistically significant.

**Table 1:** The associations between potential confounding factors and HU in multivariable-adjusted model.

Variable	Male			Female		
	Adjusted OR	95%CI	P value	Adjusted OR	95%CI	P value
Age	1.00	0.99–1.01	0.994	1.02	1.00–1.04	0.097
BMI	1.79	1.46–2.20	0.000	1.81	1.35–2.42	0.000
Smoking	0.82	0.67–1.01	0.058	1.60	0.81–3.13	0.175
Alcohol drinking	1.68	1.36–2.07	0.000	1.27	0.87–1.86	0.217
Activity level	0.98	0.95–1.01	0.176	1.01	0.97–1.05	0.678
Mean total energy intake	1.19	1.07–1.33	0.002	0.93	0.77–1.12	0.451
Mean fiber intake	1.17	1.01–1.35	0.041	1.11	0.90–1.35	0.328
Nutrients supplementation	1.00	0.80–1.24	0.984	0.83	0.62–1.12	0.219
Educational level	1.07	0.88–1.31	0.509	0.96	0.71–1.30	0.774
Diabetes	0.82	0.60–1.10	0.185	0.96	0.60–1.55	0.868
Hypertension	1.40	1.14–1.72	0.001	1.91	1.41–2.59	0.000
HDL-cholesterol	0.62	0.45–0.86	0.004	0.36	0.23–0.57	0.000
LDL-cholesterol	1.10	0.99–1.22	0.079	1.14	0.98–1.33	0.083
Triglyceride	1.12	1.07–1.17	0.000	1.10	1.01–1.19	0.030
Creatinine	1.04	1.03–1.04	0.000	1.04	1.03–1.05	0.000

## Results

The characteristics of the study population in terms of the quintiles of the total dietary Mg intake are shown in Table 2. For the Chinese population, significant differences were observed across all quintiles of Mg intake for age, gender, BMI, smoking status, alcohol drinking status, activity level, mean total energy intake, mean fiber intake, nutrients supplementation, education level, diabetes, hypertension, serum creatinine, HDL-cholesterol and TG. No clear unadjusted association was observed between Mg intake and the hypertension and LDL-cholesterol.

**Table 2:** Characteristics among 5168 participants according to quintiles of total Mg intake. Data are mean (Standard Deviation), unless otherwise indicated; Mg, magnesium.

Characteristics	Quintiles of Mg intake					
	1 (lowest)	2	3	4	5 (highest)	*P* ^#^
Median Mg intake (mg/d)	165.5	253.4	335.3	454.2	644.1	-
Age (years)	53.7 (7.8)	52.9 (7.6)	52.6 (7.3)	53.2 (7.8)	52.9 (7.4)	0.035
Male (%)	36.9	49.0	53.6	58.2	63.3	0.000
BMI (kg/m2)	23.9 (3.3)	24.3 (3.3)	24.6 (3.1)	24.6 (3.1)	25.0 (3.1)	0.000
Smoking (%)	14.8	21.2	20.7	27.1	28.4	0.000
Alcohol drinking (%)	22.8	32.2	37.8	40.5	45.3	0.000
Activity level (h/w)	2.2 (3.7)	2.2 (3.6)	2.2 (3.4)	2.4 (3.5)	2.4 (3.5)	0.000
Mean total energy intake (kcal/d)	986.6 (322.7)	1324.2 (345.7)	1571.1 (412.9)	1840.8 (474.8)	2524.4 (1040.4)	0.000
Mean fiber intake (g/d)	5.9 (2.6)	10.7 (3.7)	15.1 (4.8)	23.1 (7.0)	37.2 (17.5)	0.000
Nutrients supplementation (%)	27.1	33.3	35.7	39.3	39.3	0.000
High school background or above (%)	33.5	44.9	50.1	51.6	55.3	0.000
Diabetes (%)	7.9	8.9	9.0	10.8	11.9	0.015
Hypertension (%)	29.8	33.5	32.9	34.6	34.2	0.152
HDL-cholesterol (mmol/L)	1.6 (0.4)	1.5 (0.4)	1.5 (0.4)	1.5 (0.4)	1.5 (0.4)	0.000
LDL-cholesterol (mmol/L)	2.9 (0.9)	3.0 (0.9)	3.0 (0.9)	3.0 (0.9)	2.9 (0.9)	0.089
Triglyceride (mmol/L)	1.8 (1.7)	1.8 (1.7)	1.9 (1.7)	1.9 (1.8)	2.0 (2.0)	0.000
Creatinine (μmol/L)	83.3 (31.2)	85.0 (48.9)	84.3 (19.8)	85.4 (19.7)	86.4 (19.1)	0.000
HU (%)						
Male	22.3	23.3	22.4	22.9	23.3	0.794
Female	9.7	10.2	10.2	7.6	12.7	0.537
Uric acid (μmol/L)						
Male	358.4 (89.1)	360.0 (81.6)	362.3 (80.3)	366.6 (81.4)	360.6 (78.9)	0.542
Female	274.3 (66.5)	272.5 (67.8)	276.5 (67.6)	275.6 (62.2)	285.1 (68.6)	0.055

^#^ *P* values are for test of difference across all quintiles of Mg intake.

The overall prevalence of HU among the subjects of this cross-sectional study (40 to 85 years old, average age = 50.1±7.6) was 16.7%. A significant association between Mg intake and HU was observed in the multi-variable model which was adjusted by age, gender, BMI, smoking status, alcohol drinking status, activity level, mean total energy intake, mean fiber intake, nutrients supplementation, education level, diabetes, hypertension, serum creatinine, HDL-cholesterol, LDL-cholesterol and TG, shown in Table 3. The relative odds of HU were decreased by 0.57 times in the fourth quintile of Mg intake (OR 0.57, 95% CI 0.35–0.94) and 0.55 times in the fifth quintile (OR 0.55, 95% CI 0.30–1.01) comparing with the lowest quintile, and *P* for trend was 0.091. The results of multivariable linear regression also suggested a significant inverse association between serum uric acid and Mg intake (β = -0.028, P = 0.022).

**Table 3:** Multivariable-adjusted relations of dietary of Mg intake and HU (n = 5168). Data are adjusted OR (95% CI), unless otherwise indicated; Mg, magnesium; n, number; HU, hyperuricemia.

	Quintiles of Mg intake					
	1 (lowest)	2	3	4	5 (highest)	*P* for trend
Total population (n = 5168)						
Mg intake (mg/d)	165.5	253.4	335.3	454.2	644.1	-
Participants (n)	1033	1034	1034	1035	1032	-
Multi-variable adjusted ORs	1.00 (Reference)	0.90 (0.67–1.21)	0.74 (0.50–1.08)	0.57 (0.35–0.94)	0.55 (0.30–1.01)	0.091
*P* values	-	0.489	0.115	0.027	0.052	-
Male (n = 2697)						
Mg intake (mg/d)	185.4	281.0	367.2	491.1	662.1	-
Participants (n)	539	540	539	540	539	-
Multi-variable adjusted ORs	1.00 (Reference)	0.74 (0.52–1.05)	0.62 (0.40–0.97)	0.40 (0.23–0.72)	0.35 (0.17–0.71)	0.006
*P* values	-	0.088	0.035	0.002	0.004	-
Female (n = 2471)						
Mg intake (mg/d)	147.0	223.9	304.7	405.8	616.0	-
Participants (n)	494	494	495	494	494	-
Multi-variable adjusted ORs	1.00 (Reference)	1.10 (0.65–1.88)	1.43 (0.72–2.81)	0.87 (0.36–2.14)	1.11 (0.37–3.36)	0.896
*P* values	-	0.719	0.304	0.765	0.853	-

*Multi-variable model was adjusted for age, BMI, gender, educational level, activity level, total energy intake, fiber intake, smoking status, alcohol drinking status, nutrients supplementation, diabetes, hypertension, serum creatinine, HDL-cholesterol, LDL-cholesterol and triglyceride.

For male, the prevalence of HU was 22.9% in this study. Table 3 presents the multi-variable adjusted associations between HU and dietary Mg intake. The relative odds of HU were decreased by 0.62 times in the third quintile of Mg intake (OR 0.62, 95% CI 0.40–0.97), 0.40 times in the fourth quintile (OR 0.40, 95% CI 0.23–0.72) and 0.35 times in the fifth quintile (OR 0.35, 95% CI 0.17–0.71) comparing with the lowest quintile, and *P* for trend was 0.006. Multivariable adjusted inverse association was also existed between serum uric acid and Mg intake in male population (β = -0.061, P = 0.002).

For female, the prevalence of HU was 10.0% in this study. Significant association between Mg intake and HU was rejected by the multi-variable model. The multivariable-adjusted ORs (95% CI) of HU across the five quintiles of Mg intake were 1, 1.10 (95% CI 0.65–1.88), 1.43 (95% CI 0.72–2.81), 0.87 (95% CI 0.36–2.14) and 1.11 (95% CI 0.37–3.36) respectively, while *P* for trend was 0.896. There was not a significant association between serum uric acid and Mg intake in female population too ((β = 0.006, P = 0.707).

## Discussion

This cross-sectional study observed a negative association between dietary Mg intake and HU, independent of some major confounding factors. In addition, this assiciation remained valid for male, but not for female. To our best knowledge, this is the first study that confirmed the association between dietary Mg intake and HU.

Observational data indicated that HU has a high prevalence among people with an increased intake of meat, seafood, soft drinks, and fructose [30–32]. On the contrary, emerging data also revealed that dietary interventions, such as the intake of dairy milk and vitamin C, might be beneficial for the prevention and management of HU [33,34]. The findings of this cross-sectional study comes to a conclusion that dietary Mg intake is inversely associated with HU, supporting a potential role of Mg in the prevention of HU. A similar finding was also reported in Navin’s study [26], which suggested a negative association between the serum Mg level and the increased uric acid concentration among 94 diabetic retinopathy patients.

To calculate the adjusted OR of each quintile of Mg intake, the multivariable model in this study was adjusted for a considerable number of potentially confounding factors. We found some other significant factors associated with HU, such as serum creatinine, BMI, TG, HDL-cholesterol, LDL-cholesterol and hypertension. Uric acid has been proven to be a maker of renal failure [35], it seems certain that HU is related to serum creatinine levels which are usually measured to assess the overall kidney function. Previous studies also indicated that HU often occured in association with obesity [36,37]. Furthermore, Noone et al. [38] observed that HU is associated with hypertension in pediatric patients with chronic kidney disease. These provided internal validation of this population to some extent.

This cross-sectional study mainly indicated an inverse association between dietary Mg intake and HU. Although the kidney is responsible for 60–70% of total body uric acid excretion, the remaining uric acid excretion takes place via the gastrointestinal tract [39]. Mg, as a laxative [40], plays a potential role to increase the excretion of uric acid. Several previous studies also revealed that a lower Mg intake was associated with a higher CRP level, which is the most sensitive biomarker for inflammation [18–22]. Recently, Dibaba DT et al. [18] conducted a meta-analysis and systematic review covering seven cross-sectional studies, the findings of which confirmed the aforementioned association. In addition, it has been reported that serum uric acid was positively associated with some inflammatory cytokines, such as interleukin-6 (IL-6) and tumor necrosis factor-a (TNF-a) [41–43]. Furthermore, an increased level of serum uric acid could contribute to inflammatory arthritis when it was deposited as crystals in joints [44,45]. From the analysis above, it is speculated that dietary Mg intake maybe negatively associated with the serum uric acid level through the inflammatory mechanism.

An interesting finding of the present study is that the inverse association between dietary Mg intake and HU is only valid for male, but not for female. One possible explanation is that the lower serum estrogen level in middle aged and old women (menopausal and postmenopausal period) was found to be associated with the higher Mg level in human bodies [46–48]. Thus, the effect of dietary Mg intake on the actual Mg level in human body may be mitigated in the female population (mean age, 50.1±7.6 years).

The present study has several strengths. Firstly, this is the first study conducted on a large sample (5168 subjects) that directly relates dietary Mg intake to HU. Secondly, the multivariable model was adjusted for a considerable number of potentially confounding factors, such as diabetes, hypertension, and blood lipid levels, which greatly improved the reliability of the results. Thirdly, this study adopted FFQ, an effective measurement method for the dietary micronutrient intake [49,50], and measured dietary intake in the past one year. It might better represent the average level of micronutrients compared to the measurement of instant serum level.

Limitations of the present study should also be acknowledged. The cross-sectional design of this study precluded causal correlations, and thus, further prospective studies and intervention trials should be undertaken to establish a causal association between dietary Mg intake and HU. Since no previous research investigated the association between dietary Mg intake and HU, the value of this study should not be blotted out due to the cross-sectional nature. Another limitation lies in the FFQ method itself, which is a relatively less accurate method for reflecting the instant level of micronutrients comparing with the blood concentration measurement. However, blood level may not fully reflect the nutritional status [51], and it is also difficult to reflect the accurate circulating level during a certain period of time. Meanwhile, the serum Mg level only represents less than 1% of the total body Mg, so it is not a good predictor for the total body Mg content [52,53]. Finally, further studies are needed to explore the mechanism of this correlation.

## Conclusions

The findings of this cross-sectional study indicated that dietary Mg intake is inversely associated with HU, independent of some major confounding factors. In addition, this association remains valid for the male subgroup, but not for the female subgroup.

## Supporting Information

S1 File STROBE Statement. (PDF)
